# Legal Notes for Institutional Workers

**Published:** 1914-06-13

**Authors:** 


### LEGAL NOTES FOR INSTITUTIONAL WORKERS.

### Climatic Conditions as an "Accident."

When the law of workmen's compensation was introduced into this country in 1897 it was assumed generally that the expression in the statute "personal injury by accident" meant something in the nature of a blow or cut, or bodily wound of some kind, and it came as a distinct surprise when the House of Lords laid down that the contracting of a disease by a workman without any other form of bodily injury might ground a claim for compensation. When the law was revised and re-enacted in 1906 that decision was one of the causes which led to the insertion in the Act of 1906 of provisions whereby the benefits conferred by that Act are specifically made applicable to "industrial diseases." That being so, it might be thought that the suffering of any disease not expressly included among the statutory industrial diseases would not entitle a workman to compensation, but several decisions of the House of Lords and Court of Appeal have proved that that also is a misconception. The latest of these, *Brown* v. *J. Watson, Limited*, is an illustration of this. An accident occurred that interfered with the regular working of a mine and one of the employees was consequently exposed to rigorous climatic conditions for a prolonged period, and thereby contracted the disease (pneumonia) from which he died. The Court held that he had died from accident arising out of and in course of the employment, there being a direct chain of causation from the accident to the death, with no interrupting *nova causa* to which death could be ascribed. The case is certainly an advance on former decisions, and a decided extension of the benefits of the Act.

### Is a Doctor a Hospital Servant?

The case of *Hillyer* v. *Saint Bartholomew's Hospital*, which established the rule that a doctor is not the servant of a hospital in which he may be working, so as to infer damages against the hospital in the event of injury to the patient by the carelessness of the doctor, has been followed by the New York Court of Appeal. An operation was performed in the New York Hospital, which is maintained as a charitable institution. A patient brought a suit against the hospital averring that she, having agreed to an ether examination only, was, while under the influence of the ether, operated on, and that she suffered serious consequences from the operation. The Court dismissed the case, holding that the relation of master and servant did not exist between the hospital and its physicians. "The wrong was not that of the hospital. It was (if plaintiff's narrative be credited) that of the physicians, who were not defendants' servants, but were pursuing an independent calling."

### Consent to an Operation.

A separate ground of claim by the plaintiff against the hospital was that she had informed a nurse that she had not consented to an operation; and with reference to that averment the judgment of the Court was that in treating a patient nurses as well as physicians were not acting as servants of the hospital. "The superintendent is a servant of the hospital, the assistant superintendent, the orderlies, and the other members of the administrative staff are servants of the hospital; but nurses are employed to carry out the orders of the physicians. The hospital undertakes to procure for the patient the services of a nurse. It does not undertake through the agency of the nurses to render those services itself."

### Hospitals' Responsibility for Staff Negligence.

It is now well established in English law that a hospital is not liable for the wrongs done by its physicians, but English law has not yet gone the length of holding, as seems to have happened in New York, that neither is a hospital liable for the negligence of its nurses. When the case does arise, however, it will certainly be keenly contested, for such a protection to hospitals would be most valuable, and the decision of the New York Court may, for what it is worth, be referred to.

An American journal, in commenting on the case, remarks that hospitals ought to have some measure of responsibility in the selection of physicians and the calling in of nurses unless all of these are chosen by the patients. As regards nurses, the question has not arisen in England, but as regards doctors the law does put a responsibility on the hospital. It does not go the length of making the hospital liable for the acts of the doctor as if he were the servant of the hospital; in fact, the law expressly rejects that idea; but it does impose on the hospital the duty of seeing that the doctors who are connected with it are of repute and competent for their work, though if that duty is fulfilled there is no further ground of liability of the hospital to a patient in it.

---

Lincoln.—The Guardians have decided to abandon the idea of building an infirmary on the site they had purchased on Burton Road, and to adopt plans prepared by their architect for an infirmary on Carline Road, estimated to cost £16,000.

Newtown.—In consequence of the lowest tender for the proposed new tuberculosis hospital at Newtown working out at considerably more per bed than the Welsh Commissioners could sanction, the Montgomeryshire Sanatorium Committee have appointed a sub-committee to discuss terms with the Guardians for the adoption of the Machynlleth Workhouse as a tuberculosis hospital and with the owner of Maesmawr Hall, Welshpool, whilst the architect has been instructed to try to reduce the cost of the proposed new hospital at Newtown.

